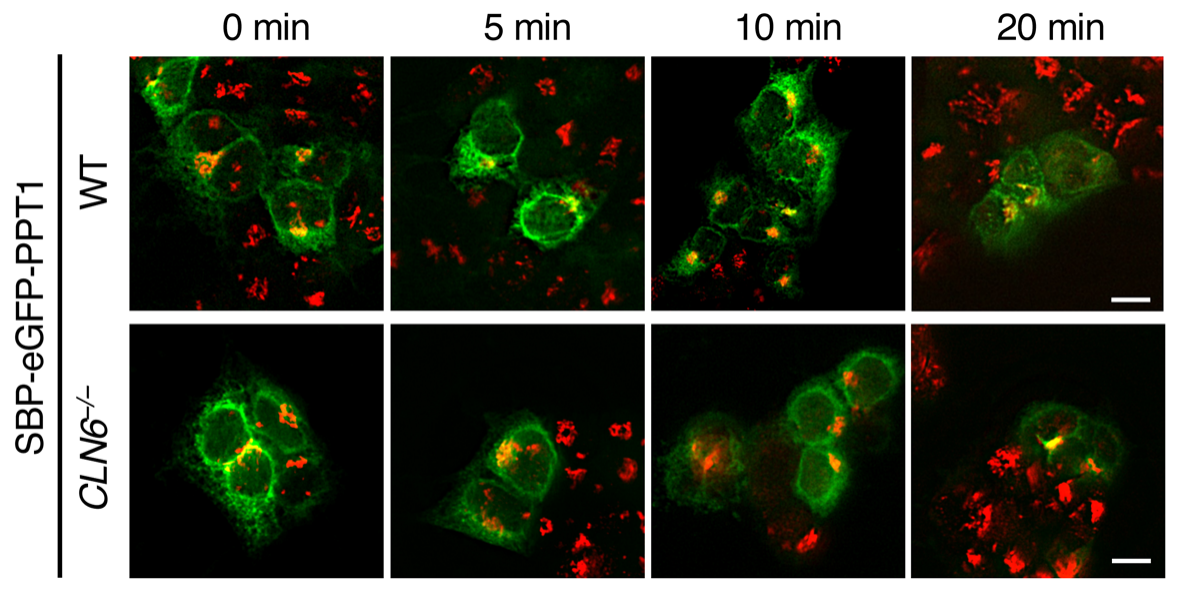# Corrigendum to A CLN6-CLN8 complex recruits lysosomal enzymes at the ER for Golgi transfer

**DOI:** 10.1172/JCI202081

**Published:** 2025-12-15

**Authors:** Lakshya Bajaj, Jaiprakash Sharma, Alberto di Ronza, Pengcheng Zhang, Aiden Eblimit, Rituraj Pal, Dany Roman, John R. Collette, Clarissa Booth, Kevin T. Chang, Richard N. Sifers, Sung Y. Jung, Jill M. Weimer, Rui Chen, Randy W. Schekman, Marco Sardiello

Original citation: *J Clin Invest*. 2020;130(8):4118–4132. https://doi.org/10.1172/JCI130955

Citation for this corrigendum: *J Clin Invest*. 2025;135(24):e202081. https://doi.org/10.1172/JCI202081

In Figure 2A of the original article, the CLN6-Y1 Y2-LMF1 image was incorrect and was derived from the same image as 4D CLN6-Y1 CLN6-Y1. Additionally, in Figure 2A, the CLN6-Y1 Y2-CLN8 image was incorrect and was a duplicate of Figure 5A CLN6ΔL2-Y1 CLN6-Y2 image. An updated Figure 2A, CLN6-Y1 Y2-LMF1 image is provided to show data from a consistent experiment and a consistent magnification. In Figure 5A, the CLN6ΔL2-Y1 CLN6-Y2 image and CLN6ΔL-Y1 CLN6ΔL2-Y2 were swapped. Lastly, in Figure 7A, the SBP-eGFP-PPT1/WT 20 min image was incorrect and was a duplicate of the LyzC SBP-eGFP WT rightmost image in Supplemental Figure 7. The corrected figure panels, based on the original source data, are provided below. The HTML and PDF versions of the paper have been updated.

The authors regret the errors.

## Figures and Tables

**Figure 2:**
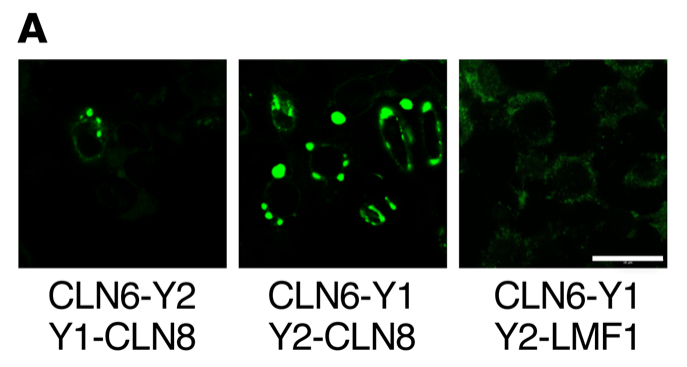


**Figure 5:**
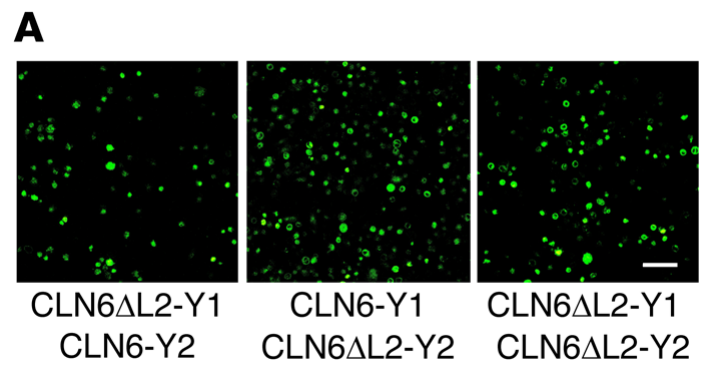


**Figure 7:**